# Perception of solar UV radiation by plants: photoreceptors and mechanisms

**DOI:** 10.1093/plphys/kiab162

**Published:** 2021-04-07

**Authors:** Neha Rai, Luis Orlando Morales, Pedro José Aphalo

**Affiliations:** 1 Organismal and Evolutionary Biology, Viikki Plant Science Center (ViPS), Faculty of Biological and Environmental Sciences, University of Helsinki, 00014 Helsinki, Finland; 2 School of Science and Technology, The Life Science Center-Biology, Örebro University, SE-70182 Örebro, Sweden

## Abstract

About 95% of the ultraviolet (UV) photons reaching the Earth’s surface are UV-A (315–400 nm) photons. Plant responses to UV-A radiation have been less frequently studied than those to UV-B (280–315 nm) radiation. Most previous studies on UV-A radiation have used an unrealistic balance between UV-A, UV-B, and photosynthetically active radiation (PAR). Consequently, results from these studies are difficult to interpret from an ecological perspective, leaving an important gap in our understanding of the perception of solar UV radiation by plants. Previously, it was assumed UV-A/blue photoreceptors, cryptochromes and phototropins mediated photomorphogenic responses to UV-A radiation and “UV-B photoreceptor” UV RESISTANCE LOCUS 8 (UVR8) to UV-B radiation. However, our understanding of how UV-A radiation is perceived by plants has recently improved. Experiments using a realistic balance between UV-B, UV-A, and PAR have demonstrated that UVR8 can play a major role in the perception of both UV-B and short-wavelength UV-A (UV-A_sw_, 315 to ∼350 nm) radiation. These experiments also showed that UVR8 and cryptochromes jointly regulate gene expression through interactions that alter the relative sensitivity to UV-B, UV-A, and blue wavelengths. Negative feedback loops on the action of these photoreceptors can arise from gene expression, signaling crosstalk, and absorption of UV photons by phenolic metabolites. These interactions explain why exposure to blue light modulates photomorphogenic responses to UV-B and UV-A_sw_ radiation. Future studies will need to distinguish between short and long wavelengths of UV-A radiation and to consider UVR8’s role as a UV-B/UV-A_sw_ photoreceptor in sunlight.

## Teaser

In sunlight, UVR8 mediates the perception of both UV-B and short-wavelength UV-A radiation with its sensitivity moderated by blue light perceived through cryptochromes.

## Introduction

Ultraviolet (UV) radiation (100–400 nm) is divided based on wavelength into UV-C (100–280 nm), UV-B (280–315 nm), and UV-A (315–400 nm) bands. These definitions originate from discussions held in 1932 and were later used for CIE and ISO standards (Björn, 2015). Wavelength limits were likely chosen based on the properties of DNA and ozone, and available instrumentation, without consideration of plant responses. Despite this, these limits have been used with only small variations almost unquestioned in plant research for nearly a century. This is in stark contrast to the definition of photosynthetically active radiation (PAR, 400–700 nm) that is based on measured action spectra (McCree, 1972).

**Figure 1:**
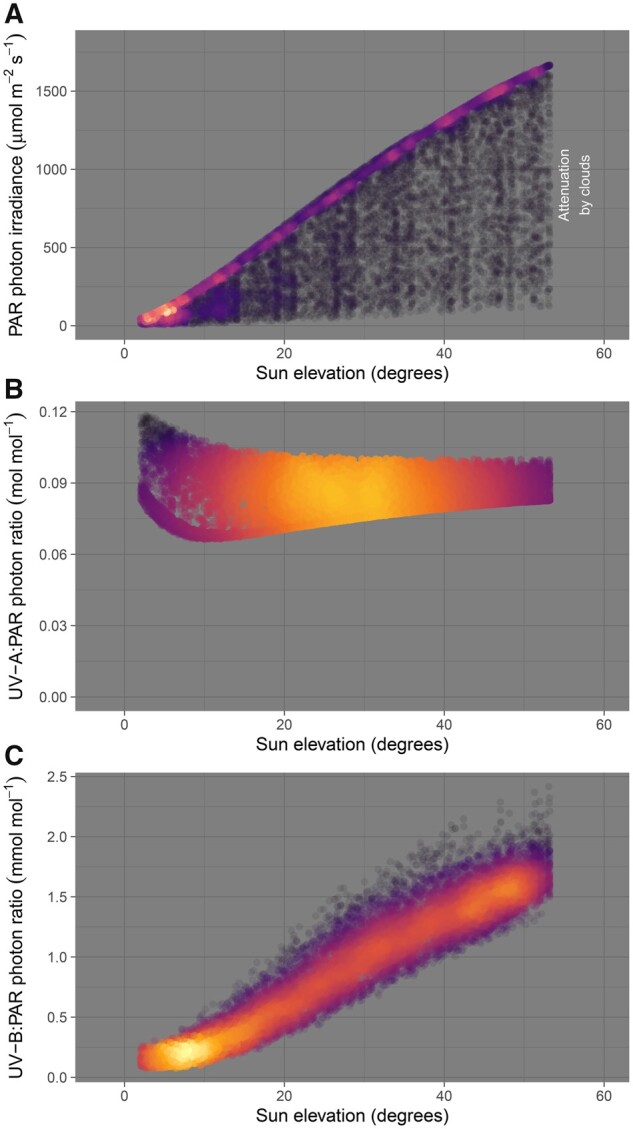
Sunlight during five summers at Kumpula, Helsinki (60.20400 N, 24.95811 E). Summaries computed from simulated hourly spectral solar irradiance at ground level are plotted against solar elevation above the horizon. A, Photon irradiance of PAR; B, UV-A:PAR photon ratio; C, UV-B:PAR photon ratio. The color indicates the local density of observations, with the “hotter” red to yellow regions mostly corresponding to data for clear-sky conditions and the “cooler” dark points corresponding to different degrees of cloud cover. Original data consist in 11,759 hourly simulations for sun elevation angles higher than 3–7 degrees at the center of the hour, for the period 1 May to 30 September of years 2013–2017, produced by Anders V. Lindfors with a radiation transfer model (libradtran) (Lindfors et al., 2009; Emde et al., 2016).

In sunlight, the photon ratio between UV radiation and PAR is close to 0.1. Extraterrestrial UV-C and UV-B radiation of wavelength < 290 nm is absorbed in the atmosphere and more than 95% of the UV photon irradiance reaching the Earth’s surface falls within the UV-A region. The UV-A:PAR ratio is less affected by the length of the path through the atmosphere than the UV-B:PAR ratio and therefore it varies much less with sun elevation (Figure 1). Both ratios are, in turn, less affected by clouds than PAR irradiance itself (Figure 1).

**ADVANCES**

- The “UV-B” photoreceptor UVR8 mediates perception of both UV-A and UV-B radiation in sunlight.
- Short and long wavelengths within the UV-A waveband are perceived through UVR8 and CRYs, respectively.
- CRYs-dependent signaling drastically downregulates UVR8-mediated responses to UV-B and short-wavelength UV-A radiation.
- Redundancy in photoreceptor function ensures tolerance of exposure to solar UV radiation, allowing survival of *uvr8* and *cry1cry2* mutants.
- Multiple negative feedback loops downstream of UVR8 and CRYs moderate the responses they mediate and make possible the convergence of these responses towards steady states.

The detection of thinning of the ozone layer due to human activity nearly 40 years ago triggered strong interest in UV-B radiation as a possible stressor for plants. However, the current view is that solar UV radiation acts mainly as a regulator of growth and development of plants and only exceptionally as a stressor (Jenkins, 2017; Verdaguer et al., 2017). In UV-acclimated plants, the regulatory effect of UV radiation is predominantly mediated by photoreceptors or light-sensing pigments, while damage is avoided or readily repaired (Robson et al., 2019). However, with high UV-B doses, DNA damage itself can trigger regulatory responses (Dotto and Casati, 2017).

UV RESISTANCE LOCUS 8 (UVR8) is commonly described as a UV-B photoreceptor (Rizzini et al., 2011) while cryptochromes 1 and 2 (cry1, cry2, CRYs when referring to both), phototropins 1 and 2 (phot1, phot2, PHOTs), and three zeitlupe proteins are described as UV-A/blue photoreceptors (Briggs and Huala, 1999; Christie et al., 2015). These roles have been attributed to UVR8, CRYs, and PHOTs based on their strong photon absorption in these regions (Briggs and Christie, 2002; Banerjee et al., 2007; Christie et al., 2012), and on the responses to monochromatic radiation that they mediate (Rizzini et al., 2011; Christie et al., 2015; Jenkins, 2017; Robson et al., 2019). However, information on the role of photoreceptors in plant responses to UV-A radiation has been scarce. Most past studies on the function of CRYs and PHOTs have focused only on blue-light-induced responses, likely because of an expectation that the mechanism depends on the photoreceptor rather than on the wavelength. Similarly, most studies on the function of UVR8 have focused on UV-B. In addition, the role of cryptochrome 3 or cry-dash in perception and signaling of UV-A radiation and blue light remains unclear (Chaves et al., 2011). This had left a gap in our knowledge of photoreceptor-dependent responses to UV radiation. This gap was most notable for photoreceptor function in sunlight and shade light, as the artificial lighting used in most controlled-environment experiments has been very different in its spectrum and irradiance from those in the natural environment.

In this article, we review recent advances in our understanding of the role of photoreceptors in plant responses to solar UV radiation. We discuss how the action of photoreceptors depends on the shape of the solar spectrum and how responses are dependent on the joint action of photoreceptors. With future research in mind, we highlight the current challenges faced by research on plants’ responses to solar UV radiation and suggest ways of addressing them. Whole-plant responses to UV-B and UV-A radiation (Jenkins, 2017; Verdaguer et al., 2017; Jansen and Urban, 2019), responses not mediated by photoreceptors (Hideg et al., 2013; Jenkins, 2017), as well as details of the perception of UV-B radiation (Jenkins, 2017; Yin and Ulm, 2017), already covered by recent reviews are beyond the scope of this update.

## Photoreceptors and responses

The role of CRYs, PHOTs, and zeitlupe proteins in the regulation of multiple plant responses to blue light has been well demonstrated and reviewed (Briggs and Christie, 2002; Chen et al., 2004; Yu et al., 2010; Christie et al., 2015; Yang et al., 2017). CRYs mediate most blue-light-induced changes in gene expression, as well as cotyledon expansion, accumulation of phenolic metabolites, and regulation of flowering time (Kleine et al., 2007; Yu et al., 2010; Christie et al., 2015; Wang et al., 2017). PHOTs mediate phototropism and chloroplast movement (Briggs and Christie, 2002; Christie et al., 2015), whereas both CRYs and PHOTs mediate hypocotyl-growth inhibition and stomatal opening under blue light (Folta and Spalding, 2001; Wang et al., 2020).

Similarly, the role of UVR8 in the perception of UV-B radiation has been clearly demonstrated and reviewed (Tilbrook et al., 2013; Jenkins, 2014; Jenkins, 2017; Yin and Ulm, 2017). UVR8 mediates UV-B-induced changes in gene expression and photomorphogenic responses such as inhibition of hypocotyl elongation, promotion of cotyledon expansion, induction of flavonoid biosynthesis, and accumulation of flavonoid compounds (Brown et al., 2005; Favory et al., 2009; Demkura and Ballaré, 2012; Morales et al., 2013; Jenkins, 2017; Yin and Ulm, 2017; Rai et al., 2019). UVR8 is required for UV-B-induced phototropism of the Arabidopsis (*Arabidopsis thaliana*) inflorescence (Vanhaelewyn et al., 2019), the down-regulation of growth-related genes by UV-B radiation (Mazza and Ballaré, 2015), and the repression of shade avoidance by sun flecks (Moriconi et al., 2018). UVR8 also regulates UV-B-induced stomatal closure in Arabidopsis (Tossi et al., 2014). In etiolated Arabidopsis seedlings, only in the absence of PHOTs, UVR8 participates in UV-B-induced phototropism, whereas PHOTs regulate this response if present (Vandenbussche et al., 2014). Additional evidence for a role of phot1 in responses to UV-B radiation comes from a study on chloroplast movement in response to UV-B in detached Arabidopsis leaves (Hermanowicz et al., 2019). However, the mechanism by which PHOTs participate in UV-B responses is still unknown.

In comparison to UV-B radiation and blue light, fewer studies have addressed the role of plant photoreceptors in the perception of UV-A radiation (Lin et al., 1995; Liscum and Briggs, 1995; Fuglevand et al., 1996; Lin et al., 1996). Based on few studies done in controlled environments using “blacklight blue” lamps with peak of emission at 350 or 368 nm, it is known that cry1 mediates suppression of hypocotyl elongation, accumulation of anthocyanins, and induction of the flavonoid biosynthesis gene *CHALCONE SYNTHASE* (*CHS*) (Lin et al., 1995; Fuglevand et al., 1996; Lin et al., 1996), while phot1 mediates phototropism (Liscum et al., 2003). An outdoor experiment assessing responses to solar UV-A provided the first evidence for the possible involvement of UVR8 in UV-A-mediated changes in gene expression and accumulation of specific phenolic compounds (Morales et al., 2013). It was proposed that under solar PAR and UV-A irradiance, UVR8 interacted with other photoreceptors through signaling pathways to modulate UV-A responses in the presence of UV-B radiation (Morales et al., 2013).

**Figure 2:**
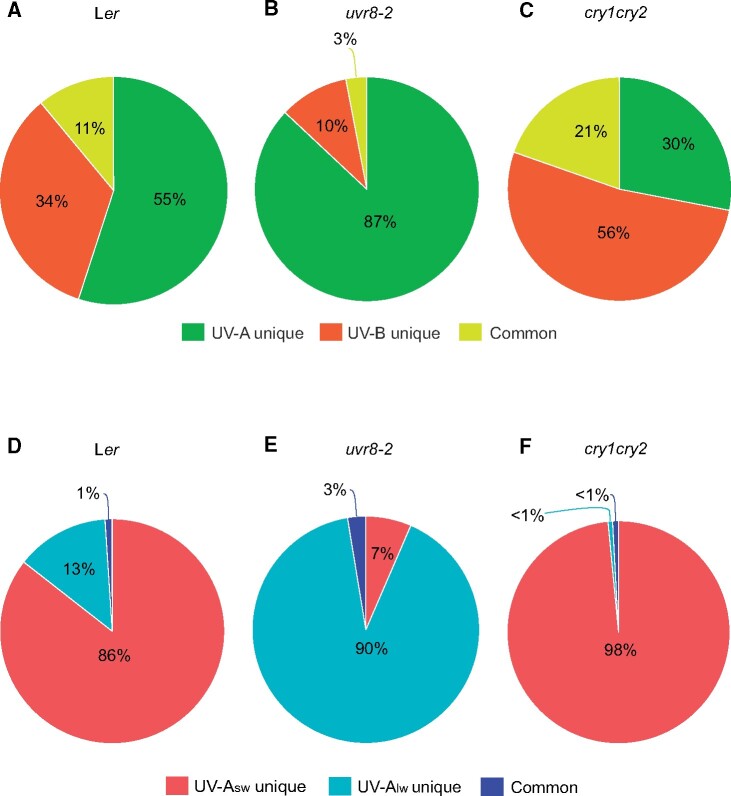
Transcript abundance after 6 h exposure to filtered sunlight in three genotypes of Arabidopsis plants. A separate pie chart for each genotype shows the percentage of differentially expressed genes responding uniquely to UV-B radiation (orange), uniquely to UV-A radiation (green), and common to both UV-B and UV-A radiation (yellow). A–C, responding uniquely to short-wavelength UV-A (UV-A_sw_) radiation (red), uniquely to long-wavelength UV-A (UV-A_lw_) radiation (light blue), and common to both UV-A_sw_ and UV-A_lw_ radiation (dark blue). D–F, Figure based on the transcriptome analysis of Rai et al. (2020).

In a recent study, using photoreceptor mutants in sunlight, Rai et al. (2020) showed that both UVR8 and CRYs mediate transcriptome-wide responses to solar UV-A (315–400 nm; Figure 2, A–C). However, within UV-A, the roles of UVR8 and CRYs differed: UVR8 was required for the responses to short-wave UV-A radiation (315–350 nm, UV-A_sw_; Figure 2, D and E), while CRYs were required for responses to long-wave UV-A radiation (350–400 nm, UV-A_lw_; Figure 2, D and F). This split at approximately 350 nm is also consistent with earlier studies reporting UVR8-independent induction of *CHS* in response to UV-A wavelengths between 350 and 400 nm (Brown et al., 2005) and UVR8-independent inhibition of stomatal opening by UV-A at 380 nm (Isner et al., 2019). Given these results, the relevance of the definition of the UV-A waveband (315–400 nm) to plants needs to be re-assessed (Box 1).

BOX 1 Photoreceptor action and the definition of the UV-A waveband

Rai et al. (2020) showed that solar UV-Asw (315–350 nm) was far more effective than solar UV-Alw (350–400 nm) in the regulation of transcript abundance across the whole transcriptome. Effectiveness was assessed based on the number of genes and the magnitude of response for selected individual genes, both of which were many times higher in UV-Asw than UV-Alw. The transcriptomic analysis also showed that different sets of genes were triggered by UV-Asw and UV-Alw in the WT, with only 1% of genes in common (Figure 2D), while the overlap was larger when considering UV-B and whole of UV-A (Figure 2A). These results indicated that in sunlight the main role of CRYs is in responses to blue light, whereas in responses to UV-A radiation their role is minor compared to that of UVR8 (Rai et al., 2020). As monomerization of UVR8 dimers driven by photon absorption is considered crucial for its action (Rizzini et al., 2011), that the purified UVR8 protein monomerizes in vitro only upon exposure to wavelengths of 335 nm or shorter, helps explain why UVR8’s action is limited to the UV-B and UV-Asw regions (Rai et al. 2020). UVR8 is an unusual plant photoreceptor where tryptophan amino acids within the UVR8 protein behave as chromophore (Christie et al., 2012), and in that excitation transfer among the three tryptophan groups, which absorb maximally at slightly different wavelengths results in enhanced quantum efficiency (Li et al., 2020). This photochemical mechanism has been considered capable of explaining the transition in UVR8 function at a wavelength near 350 nm (Li et al., 2020). Furthermore, it is possible that this cut-off wavelength observed in vitro is slightly different in vivo. Thus, the UV-A waveband can be divided into two different regions (UV-Asw and UV-Alw) based on a transition at 335 nm to 350 nm between radiation-dependent action of UVR8 and CRYs in the regulation of gene expression (Rai et al., 2020). Based on the mechanism behind the transition, the wavelength boundary observed in Arabidopsis can be expected to be similar to that in other plant species, although further studies are needed to confirm this.

CRYs may not have a direct role in responses to UV-B and UV-A_sw_ radiation, but when activated by UV-A_lw_ and PAR, CRYs negatively regulate the UVR8-mediated gene expression occurring in response to UV-B and UV-A_sw_ (Rai et al., 2019, 2020; Tissot and Ulm, 2020). The double mutant *cry1cry2* had a stronger gene-expression response to UV-B and UV-A_sw_ radiation than the WT (Figure 2, D and F; Rai et al., 2019, 2020; Tissot and Ulm, 2020). Furthermore, Tissot and Ulm (2020) dissected mechanistically the regulation of UV-B responses by CRYs with an involvement of REPRESSOR OF UV-B PHOTOMORPHOGENESIS 1 (RUP1) and RUP2, the negative feedback regulator of UVR8 signaling (see the “Molecular mechanisms of photoreceptor action” section). Thus, although UVR8 might be the primary sensor of UV-B and UV-A_sw_ radiation, CRYs-mediated blue light signaling negatively regulates the activity of the UVR8 photoreceptor (Rai et al., 2019, 2020; Tissot and Ulm, 2020). This highlights the need for direct evidence supporting photoreceptor activation at specific wavelengths, as altered responses in photoreceptor mutants provide only circumstantial evidence due to regulatory interactions.

As growth and survival are important determinants of plants’ fitness in nature, it is relevant to understand how photoreceptors regulate these responses both in the UV-B and UV-A regions. It has been shown that UVR8, CRYs, and a red/far-red photoreceptor phytochrome B (phyB) modulate plant growth in response to UV-B, UV-A_sw_, and UV-A_lw_, in the presence of PAR (Rai et al., 2019; Tissot and Ulm, 2020). If either of UVR8 or CRYs are present, plants grew normally in response to UV-B, UV-A_sw_, and UV-A_lw_ radiation; however, when UVR8 and CRYs were simultaneously absent in mutants, growth was drastically reduced (Rai et al., 2019; Tissot and Ulm, 2020). Furthermore, CRYs and phyB act redundantly with UVR8 to provide UV-B tolerance in plants (Rai et al., 2019; Tissot and Ulm, 2020). This indicates that UVR8 and CRYs or UVR8 and phyB can substitute for each other in triggering acclimation to sunlight allowing Arabidopsis plants to grow normally. However, under harsher environmental conditions substitution between UVR8 and CRYs or UVR8 and phyB could be less effective in maintaining plants’ fitness.

## Optical phenomena

The likelihood of photons being absorbed by a photoreceptor depends both on the absorption properties of the photoreceptor and on the wavelength and number of photons impinging on it. Sensing also requires that the excitation of the photoreceptor is transduced into a downstream response. In cotyledons of Arabidopsis seedlings, expression of UVR8 in the epidermis and in the mesophyll in a UVR8 null-mutant background using tissue-specific promoters contributed to regulation of the expression of *ELONGATED HYPOCOTYL 5* (*HY5*) and *EARLY LIGHT-INDUCED PROTEIN 2* (*ELIP*2) locally within each of these tissues (Bernula et al., 2017). In the control, using the native promoter, UVR8 was expressed in the epidermis more than in the mesophyll, while not detected in the vascular tissue (Bernula et al., 2017). In leaf samples collected from plants of 42 species growing outdoors, species’ mean epidermal transmittance to UV-B radiation (300 nm) varied between less than 1% and 58% (Day et al., 1993). The spectrum and direction of the radiation incident on leaf surfaces is altered by absorption and reflection before reaching plant tissues where the photoreceptors are located (Day et al., 1993; Brodersen and Vogelmann, 2010).

Phenolic metabolites accumulated on the cuticle and in epidermal cells strongly absorb UV radiation (Krauss et al., 1997; Siipola et al., 2015) while cuticular waxes usually reflect both UV radiation and PAR (Holmes and Keiller, 2002). In plants, the accumulation of phenolic metabolites is regulated by radiation perceived through CRYs, UVR8, and phytochromes and is responsive to UV-B, UV-A, blue, red, and far-red wavelengths (Duell-Pfaff and Wellmann, 1982; Holopainen et al., 2018). In sunlight, their accumulation frequently depends predominantly on UV-B radiation (Morales et al., 2010, 2013) but occasionally predominantly on blue light (Siipola et al., 2015). Accumulation of phenolic metabolites in epidermis apparently depends to a large extent on direct exposure of each epidermis to UV radiation (Morales et al., 2011; Bidel et al., 2015; Solanki et al., 2019; Pieristè et al., 2020).

UV absorptance and reflectance determine the transmittance of the epidermis, which can adapt across generations as a result of natural selection, acclimate over several days and, in some species, even track changes in ambient solar irradiance within tens of minutes (Veit et al., 1996; Siipola et al., 2015; Barnes et al., 2016b, 2017). For example, in barley leaves, both epidermal UV-A and UV-B transmittances varied between 35% and 2% in plants subjected to different conditions including low PAR in the absence of UV-B radiation (Kolb and Pfündel, 2005). In the same species, mean epidermal UV-A transmittance decreased from 56% to 10% in the course of 1 week during acclimation to UV exposure (Klem et al., 2015). In a few species, epidermal UV-A transmittance can vary by 50% or more through the day while in other species the range of variation is much smaller (Barnes et al., 2016a, 2016b). Variation through the course of a day has been shown to require exposure to UV radiation shorter than 350 nm (Veit et al., 1996). Epidermal UV-A transmittance also varies seasonally (Pescheck and Bilger, 2019; Solanki et al., 2019). UVR8 is located both in the epidermis and mesophyll tissues where it participates in signaling, controlling the accumulation of the same phenolic metabolites that attenuate the UV radiation entering the leaf (Bidel et al., 2015). Consequently, conforming a signaling loop that could play an important role in stabilizing plant responses to UV radiation through negative feedback (Bidel et al., 2015). The screening by phenolics is much weaker in the blue waveband than in the UV waveband, and so also the gain of the negative feedback must be weaker, consequently more strongly affecting UVR8- than CRYs-dependent signaling. There is yet no direct evidence supporting this hypothesis, but it is grounded in physical principles.

**Figure 3:**
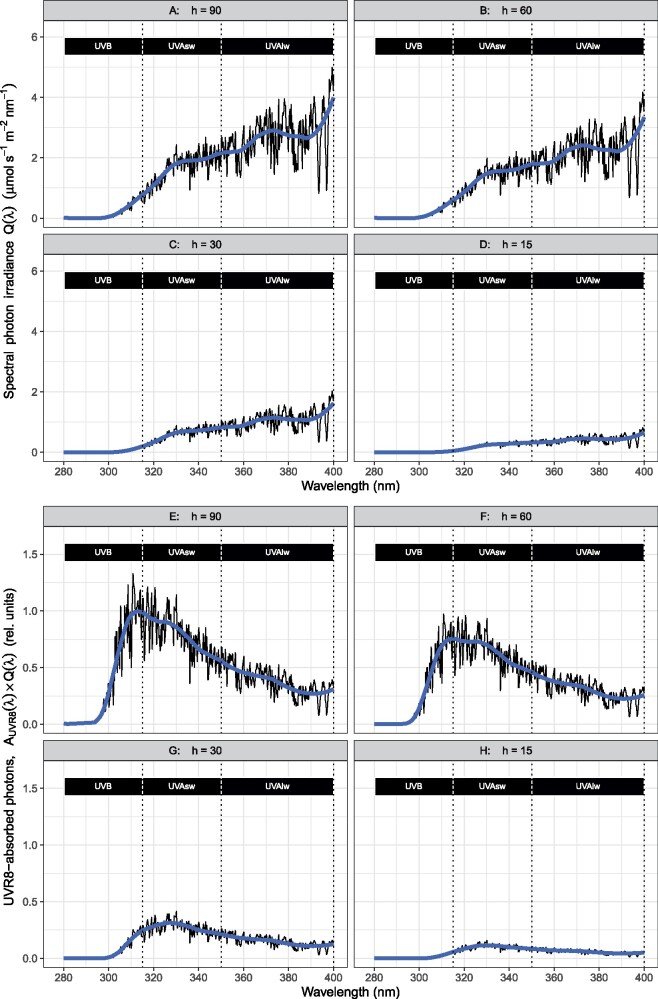
Solar UV radiation at different solar elevations and corresponding estimates of absorbed photons by photoreceptor UV RESISTANCE LOCUS 8 (UVR8) molecules. Panels (A)–(D) show modeled solar spectrum for clear sky conditions and sun elevation angles (*h*) of 90, 60, 30, and 15 degrees. Panels (E)–(H) show result from convolution of the spectra in (A)–(D) with the in vitro absorbance spectrum of the UVR8 protein (Rai et al., 2020, Supplemental Figure S7) predicting that UVR8 will absorb both UV-B and UV-A radiation in sunlight. The solar spectrum was simulated with the Quick TUV simulator for a depth of the ozone layer of 300 DU. Computations and plotting were done in R (R Core Team, 2020) with packages from the R for photobiology suite (Aphalo, 2015) and the tidyverse (Wickham et al., 2019). UV-A_sw_: short wavelength UV-A, UV-A_lw_: long wavelength UV-A.

In addition to screening by pigments, the spectrum of radiation incident on a plant determines the activation of photoreceptors. To estimate the absorption of photons by a photoreceptor in planta, we would need to know the spectral photon irradiance incident on the photoreceptor molecules and the absorption spectrum of the photoreceptor in planta. However, both quantities are difficult or impossible to measure with current methods. Notwithstanding these limitations, combining the in vitro absorption spectrum of a photoreceptor with the spectrum of radiation incident on a plant provides a crude estimate of the relative number of photons a photoreceptor could absorb at different wavelengths, as long as these wavelengths are similarly attenuated in the epidermis. By combining the in vitro absorption spectrum of the UVR8 protein, measured over the whole UV-B and UV-A regions with hourly spectral irradiances, Rai et al. (2020) showed that the steep increase in solar spectral irradiance near the boundary between UV-A and UV-B regions is enough to allow UVR8 to abundantly absorb UV-A photons, thus explaining the observed role of UVR8 in solar UV-A responses (Morales et al., 2013; Rai et al., 2020). In Figure 3, using spectral irradiance for different sun elevations above the horizon, we additionally show that UVR8 is likely to mediate the perception of UV-A_sw_ radiation both when the sun is at the zenith and the UV-B:UV-A photon ratio is at its maximum and when it is lower in the sky. This suggests that UV-A_sw_ photons perceived through UVR8 could contribute to responses to solar radiation throughout the photoperiod and at low and middle latitudes, throughout the year, that is even when solar UV-B irradiance is very weak. However, downregulation of UVR8 action by blue light absorbed by CRYs would moderate downstream responses.

**Figure 4:**
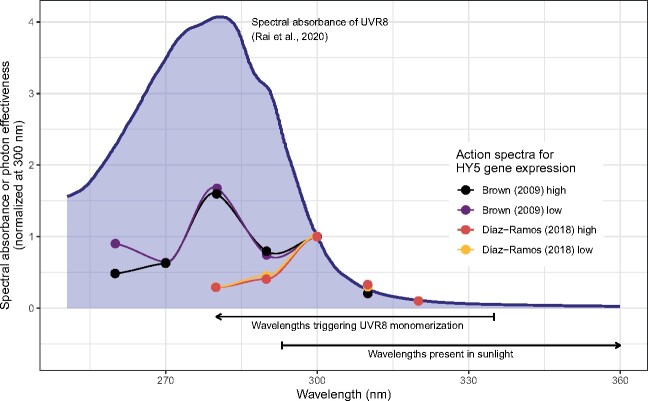
Comparison of published action spectra for UV RESISTANCE LOCUS 8 (UVR8)-mediated expression of the gene *ELONGATED HYPOCOTYL 5* (*HY5*) (Brown et al., 2009; Díaz-Ramos et al., 2018) to the published absorption spectrum of UVR8 (Rai et al., 2020). High and low refer to action spectra computed for different levels of monomerization, as reported in the original publications. All spectra are normalized to one at 300 nm.

The theoretical expectation is that in the absence of differential screening by other pigments, an action spectrum will have a shape similar to that of the absorption spectrum of the photoreceptor (Gorton, 2010). Currently available UVR8 action spectra (Brown et al., 2009; Díaz-Ramos et al., 2018), both for *HY5* expression at wavelengths 260–310/320 nm, can be scaled to match each other at wavelengths between 300 and 315 nm, and when this is done, their shape in this region resembles that of the absorption spectrum (Figure 4). However, at shorter wavelengths, the mismatch is large. Differences in the optical properties of leaves or damage caused by some wavelengths could explain the unexpectedly weak expression of *HY5* at wavelengths shorter than 290 nm, wavelengths that are anyway absent from sunlight. As the seedlings used were grown under very low light (Brown et al., 2009; Díaz-Ramos et al., 2018), a condition where accumulation of flavonoids and other UV-screening pigments is reduced (Klem et al., 2015), these action spectra could differ from those for plants growing in sunlight. As different flavonoids and phenolic acids differ in the wavelengths of maximal absorption (Kolb et al., 2005), changes in phenolic composition could also alter the shape of the action spectrum. An equivalent effect of screening by chlorophyll on the action spectra of phytochromes is well documented (Gorton, 2010) and, as discussed above, epidermal absorption in the UV region can vary widely, suggesting a similar effect on UVR8 action spectra.

Due to the interactions involving UVR8 and CRYs signaling, UVR8 action spectra measured using monochromatic light are unlikely to describe the action of UVR8 in sunlight. Polychromatic action spectra (see Cooley et al., 2000b) measured using photoreceptor mutants as controls would help disentangle the roles of UVR8 and CRYs in the perception of solar UV-A and UV-B radiation. Such an action spectrum is not yet available.

## Molecular mechanisms of photoreceptor action

The excitation mechanism of absorption of a photon by a photoreceptor is likely to be the same irrespective of wavelength. Therefore, signaling mechanisms initiated by photoreceptors have been frequently studied using a few wavelengths, for example UV-B centered at 310 to 315 nm for UVR8 and blue light centered at 430 or 460 nm for CRYs and PHOTs. These mechanisms will be discussed here in relation to their role in the perception of solar UV radiation as they have been recently reviewed from a molecular perspective (Binkert and Ulm, 2017; Hideg and Strid, 2017; Jenkins, 2017; Liscum et al., 2020; Wang and Lin, 2020).

In sunlight and shade light, multiple photoreceptors are activated simultaneously making interactions downstream of them important for whole-plant responses (Casal, 2013; Ballaré and Pierik, 2017). Changes in natural illumination are usually gradual, allowing negative feedback to work effectively. As UVR8 and CRYs signaling interact to regulate gene expression in response to UV-B and UV-A radiation and to blue light (Rai et al., 2019, 2020), responses to solar UV radiation also depend on visible light. Consequently, molecular and signaling interactions can play a crucial role in the mechanism of UV perception in plants’ natural environment.

In the absence of UV-B, UVR8 exists as a homo-dimer and is mainly present in the cytosol, whereas UV-B exposure leads to UVR8 monomerization and rapid accumulation in the nucleus (Brown et al., 2005; Kaiserli and Jenkins, 2007; Rizzini et al., 2011). More recently it was also reported that UVR8 can monomerize under wavelengths in the UV-A region (up to ∼335 nm, Rai et al., 2020) which could be explained by the transfer of excitation energy between the three groups of tryptophan amino acids in the UVR8 protein (Li et al., 2020, see Box 1). Conversion from a dimer to monomer upon exposure to UV-B (Rizzini et al., 2011), followed by mobilization of the monomers from the cytosol to the nucleus are key steps in UVR8 signaling (Kaiserli and Jenkins, 2007). Conversely, upon activation by blue light, CRYs monomers form dimers (Wang et al., 2016). Cry1 is present both in the nucleus and cytosol whereas cry2 is present mainly in the nucleus (Guo et al., 1999; Wu and Spalding, 2007; Yu et al., 2007). After UV-B exposure, UVR8 interacts with the E3 ubiquitin ligase CONSTITUTIVELY PHOTOMORPHOGENIC 1 (COP1), a repressor of photomorphogenesis in darkness (Favory et al., 2009; Podolec and Ulm, 2018; Lau et al., 2019). This binding into the UVR8–COP1 complex inhibits the repressor activity of COP1 E3 ubiquitin ligase and stabilizes the HY5 transcription factor (TF), a master regulator of gene expression (Favory et al., 2009; Huang et al., 2013; Gangappa and Botto, 2016; Podolec and Ulm, 2018). Similarly, CRYs also bind to COP1 and inhibits its E3 ubiquitin ligase activity which stabilizes HY5 (Hoecker, 2017; Holtkotte et al., 2017; Podolec and Ulm, 2018; Lau et al., 2019). As signaling downstream of UVR8 and CRYs has many components in common, multiple points of interaction can be envisaged.

**Figure 5:**
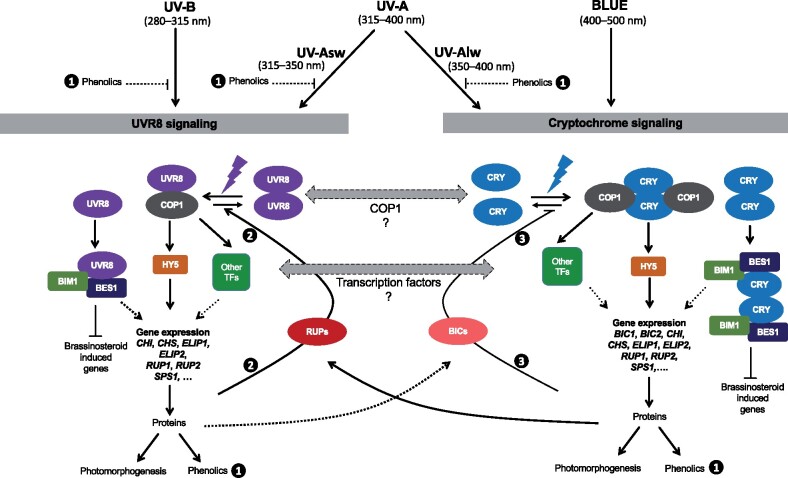
A model combining different hypotheses for coaction downstream of UV RESISTANCE LOCUS 8 (UVR8) and cryptochromes 1 and 2 (CRYs) in UV responses and possible modulation by other wavelengths. We postulate a first level of interaction through CONSTITUTIVELY PHOTOMORPHOGENIC 1 (COP1) as both UVR8 and CRYs physically interact with COP1, second level through shared TFs (e.g. ELONGATED HYPOCOTYL 5 [HY5], BRI1-EMS-SUPPRESSOR1 [BES1], BES1-INTERACTING MYC-LIKE 1 [BIM1], note that both UVR8 and CRYs physically interact with BES1 and BIM1*), and a third level through REPRESSOR OF UV-B PHOTOMORPHOGENESIS 1 (RUP1) and RUP2, and BLUE-LIGHT INHIBITOR OF CRYPTOCHROMES 1 (BIC1) and BIC2. The complete arrows show paths supported by experimental evidence while the dotted arrows show hypothetical mechanisms that are compatible with current knowledge. Numbers 1–3 refer to the negative feedback loops described in Box 2. *CHI: CHALCONE ISOMERASE*, *CHS: CHALCONE SYNTHASE*, *ELIP 1: EARLY LIGHT-INDUCED PROTEIN 1*, *ELIP2: EARLY LIGHT-INDUCED PROTEIN 2*, *SPS1: SOLANESYL DIPHOSPHATE SYNTHASE 1* (* not shown in the model).

We hypothesize that the interactions downstream of UVR8 and CRYs could take place at multiple levels, as summarized in our model (Figure 5). The first level of interaction would depend on COP1, whose WD40 domain is the site for binding with the VP-peptide motif on UVR8 and CRYs (Lau et al., 2019; Ponnu et al., 2019). However, experimental evidence for competition between the photoreceptors for binding to COP1 is still lacking. Specific TFs could operate at the second level of interaction, as both UVR8 and CRYs signaling involves some of the same TFs for the regulation of gene expression, such as HY5, BRI1-EMS-SUPPRESSOR1 (BES1), BES1-INTERACTING MYC-LIKE 1 (BIM1), and PHYTOCHROME INTERACTING FACTORs (PIFs) (Hayes et al., 2014; Gangappa and Botto, 2016; Pedmale et al., 2016; Liang et al., 2018; Wang et al., 2018). The third level of interaction could involve RUP1 and RUP2, and BLUE-LIGHT INHIBITOR OF CRYPTOCHROMES 1 (BIC1) and BIC2; which are the negative feedback regulators of UVR8 photocycle and CRYs photocycle, respectively (Gruber et al., 2010; Heijde and Ulm, 2013; Findlay and Jenkins, 2016; Wang et al., 2017). RUPs act as negative regulators of UVR8 signaling by facilitating redimerization of UVR8 monomers (Heijde and Ulm, 2013; Findlay and Jenkins, 2016), while BICs act as negative regulators of CRYs signaling by inhibiting CRYs dimerization (Wang et al., 2017). CRYs signaling activated by blue light induces *RUP1* and *RUP2* gene expression and RUP2 protein accumulation, consequently enhancing UVR8 redimerization (Tissot and Ulm, 2020). Reciprocally, UVR8 signaling activated by UV-B radiation induces *BIC1* and *BIC2*, and overexpression of BIC1 and BIC2 suppresses the CRYs-mediated UVR8 redimerization (Tissot and Ulm, 2020). Further downstream, other TFs could contribute to differential regulation of groups of genes (Rai et al., 2020). Effects of solar UV on plant-hormone signaling reflected in growth and development (Vanhaelewyn et al., 2016; Fina et al., 2017) can also give rise to interactions and feedback. Signaling can involve chained metabolic steps that by introducing delays contribute to whole-system dynamic properties (Chappell and Hahlbrock, 1984; Liao et al., 2020). In addition, we cannot yet rule out the possibility that physical interaction between the photoreceptors or other mechanisms of interaction is also involved.

The complexity of the signaling interactions downstream of UVR8 and CRYs suggests the need to study the perception of UV (and visible) radiation by plants as an integrated sensory system in which whole-system properties are largely determined by these interactions, that is whole-plant responses to sunlight cannot be predicted based solely on the responses to individual wavelengths or on the roles played by individual photoreceptors (Box 2).

BOX 2 Negative feedback and redundancy

In the model in Figure 5, we have highlighted the negative feedback loops. In any control system, negative feedback contributes to stability. However, if a step increase in an input, here radiation, occurs faster than the response of the feedback loop, the response will overshoot before stabilizing. The phenolic synthesis loop, labelled 1 in Figure 5, contributes to long-term acclimation (see section 3), and directly affects the input signal to UVR8 and CRYs as phenolics screen UV-B and UV-A radiation. A time constant of the order of one day or longer can be expected based on the rate of accumulation of flavonoids and phenolic acids (Chappell and Hahlbrock, 1984). The faster feedback affecting UVR8’s state through RUP accumulation (loop labelled 2 in Figure 5) could lead to reversible regulation of responsiveness. This agrees with the observation that upon excitation with broadband UV radiation COP1 bound to UVR8 peaks at 30 min only in non-UV-B-acclimated plants, while *HY5* transcript abundance peaks at 90 min and follows very similar time courses in both UV-B-acclimated and non-acclimated plants (Liao et al., 2020, Figures 2, 3). That the time-course of *HY5* transcript abundance does not depend on pre-exposure to UV radiation suggests that feedback can buffer downstream signaling from rapid fluctuations in photoreceptor excitation. In addition, the negative feedback on CRYs through BICs (loop labelled 3) affect CRYs signaling in a similar way as RUPs affect UVR8 signaling (Wang et al., 2017). Loops 2 and 3 have *HY5* in common and depend each on the action of both UVR8 and CRYs (Tissot and Ulm, 2020). The presence of multiple negative feedback loops and redundant signaling paths is consistent with the observed “fault-tolerance” of the sensory system: lack of either functional UVR8 or functional CRYs is not lethal in full sunlight, while the lack of both is (Rai et al., 2019; Tissot and Ulm, 2020).

## Challenges and approaches

To develop applications in plant production and conservation, the main challenge we face is to understand in depth how photomorphogenesis contributes to plant fitness and resilience, and to crop yield and quality. When studying biological systems where complex interactions prevail, one cannot expect reductionist approaches to succeed in providing a satisfactory description of phenomena (Capra and Luisi, 2014), or to significantly contribute to successful applications in agriculture (Sadras et al., 2020). The conditions under which we do experiments put strict boundaries to the range of validity of the results obtained.

As described above, the mechanism of solar UV radiation perception in Arabidopsis depends on complex interactions downstream of photoreceptors and on multiple roles for the individual photoreceptors (Casal, 2013; Morales et al., 2013; Ballaré, 2014; Rai et al., 2019, 2020; Tissot and Ulm, 2020). This suggests that plants, by combining information acquired through different photoreceptors, can differentiate wavelengths and their combinations in much more detail than it has been assumed until now. This implies that in future experiments the design of the environmental conditions used and their detailed characterization and reporting will need to be emphasized much more than in the past.

We need to also pay attention to the fact that plants’ sensory capabilities are subject to natural selection, and consequently that adaptation and acclimation of these capabilities is to be expected as for any other trait contributing to fitness (Gundel et al., 2014; Ballaré and Pierik, 2017). Differences among genotypes and species have been described, but not in the same depth of mechanistic detail as in Arabidopsis (Tossi et al., 2019). Both UVR8 and CRYs are ubiquitous in plants, while the number of copies of UVR8 and CRYs varies among species (Perrotta et al., 2001; Fernández et al., 2016). In the case of UVR8, many species have two copies, but it is not yet known if these copies differ in function (Tossi et al., 2019). Differences in same-generation- and trans-generational responses to UV radiation have been reported for *Vicia faba* accessions (Yan et al., 2019), but not the mechanisms involved. A possible mechanism could be UVR8-mediated inhibition of DNA methylation (Jiang et al., 2021). Increased emphasis on studying a breadth of species and genotypes would help in understanding the roles of UV perception in plant’s fitness in different habitats.

In experiments, UV treatments can be additive (enhancement) or subtractive (attenuation), that is use of different UV-radiation sources for different treatments versus use of different UV-absorbing filters for the different treatments (Aphalo et al., 2012). At least in principle, both approaches can be used in growth chambers, greenhouses, and outdoors. However, a UV-subtractive approach in a growth chamber is possible only if the chamber is a sun simulator, and in a greenhouse only if its cladding is UV-transparent, requirements that are very seldom fulfilled. Outdoors, UV-enhancement with lamps, results always in higher exposure than the natural one, and the maximum fractional enhancement achievable can be constrained by lamp’s output. In all cases, realistic UV treatments need not only take into account UV irradiance, but the natural balance among wavelengths in the daylight spectrum, as well as timing of UV exposure, both within the day and in relation to plant development. The key point is awareness of what is involved so as to avoid the misinterpretation of results.

The UV radiation sources most commonly used for additive treatments are special fluorescent tubes. Commercial names for these lamps can be easily misleading. Those called broad-band “UV-B lamps” can emit as much UV-A radiation as they emit UV-B radiation (Aphalo et al., 2012) while of those sold as “UV-A lamps” some emit predominantly or only UV-A_lw_ radiation, while others emit predominantly UV-A_sw_ radiation and only some types emit in both the UV-A_lw_ and UV-A_sw_ regions (Supplemental Figure S1). In the case of narrow-band UV-B lamps, the difficulties are fewer. Because of the limited radiation output of the lamps, outdoors it is currently impossible to increase solar UV-A irradiance by more than ca. 5%, while this constraint does not affect UV-B radiation supplementation (Aphalo et al., 2012). This difference stems from the lower UV-B- than UV-A irradiance in sunlight. High-power LEDs for wavelengths equal or longer than 365 nm are readily available at low cost. Those emitting at wavelengths shorter than 365 nm remain inefficient and expensive, restricting their use to the irradiation of a few plants at a time. Xenon-arc lamps although emitting a sun-like spectrum are expensive and fragile, therefore used only in small solar-simulators. Sun-simulation in growth rooms has been based on the simultaneous use of an array of different lamp types plus filters.

Given that most UV-fluorescent tubes emit over a broad range of wavelengths, they need to be used together with UV-absorbing filters to create pairs of treatments and controls differing only in the wavelengths of interest. The enhanced UV-B treatment in outdoor supplementation studies is compared with the misnamed “UV-A control,” which uses the same UV-B lamps but filtered to block UV-B radiation (Middleton and Teramura, 1993; Newsham et al., 1996). The evidence these controls provide for or against UV-A radiation effects is very weak, as these experiments have lacked a control with energized lamps filtered to remove both UV-B and UV-A radiation, which is needed to distinguish the effect of the small UV-A radiation enhancement from other side effects of the lamps. As the daily UV-A enhancement in these controls has been 0.5%–2% of solar UV-A irradiance (Cooley et al., 2000a; Tegelberg et al., 2001), it has seemed unwarranted to assume that this very small enhancement can explain the effect of the filtered UV-B lamps (Newsham et al., 1996). In spite of these limitations, some studies have wrongly interpreted the difference in plant responses between these “UV-A controls” and a control with lamps switched off as demonstrating an effect of UV-A radiation (e.g. Tegelberg et al., 2002; Bernal et al., 2015). On the other hand, the rather consistently observed responses of growth and morphology under UV-B lamps filtered to remove UV-B radiation have remained puzzling since they were discussed in detail by Cooley et al. (2000a). Future UV-A supplementation experiments using lamps and including all the necessary controls could help unravel the drivers behind plant responses observed in “UV-A controls” (Verdaguer et al., 2017), which could be mediated by UV-A_sw_ (Supplemental Figure S1, PET).

Studying responses to UV radiation in field experiments lasting months is challenging due to the variability of weather conditions including cloudiness and solar irradiance that makes replication in time desirable. A middle-ground approach between unrealistic controlled environment experiments and field experiments is the use of sun simulators in which radiation can mimic natural sunlight while controlling other environmental factors such as temperature, humidity, and wind (Rai et al., 2019). An additional approach useful to study transient and short-term responses to solar radiation is to grow plants indoors but to apply treatments outdoors using UV exclusion filters in sunlight (Morales et al., 2013, 2015; Coffey et al., 2017; Rai et al., 2020). It is also important to compare responses to UV exposure in plants grown in the absence of UV radiation with those in plants grown under UV treatments applied continuously, for example since germination, as responses can differ markedly (Rai et al., 2019). Restrictions imposed by regulations on the cultivation of transgenic plants outdoors create difficulties for field experiments resulting in delays and expenses that depend on the country where the research is done (MacKelprang and Lemaux, 2020).

While we need research done under ecologically relevant conditions and aiming at answering ecological questions, such research is greatly facilitated by the knowledge of molecular mechanisms and signaling networks obtained in laboratory experiments. Photobiological research in well-designed artificial contexts is very efficient at identifying molecular players, regulation mechanisms, and points of interaction, while understanding how regulatory interactions and signaling contribute to fitness or crop performance can only be assessed in a realistic environmental context.

Once we take into account that epigenetics, plant hormones, epidermal screening, growth, and morphology are all affected by photoreceptor-mediated responses, the number of possible mechanisms of regulation and paths for interaction and feedback grows dramatically, involving even optics of plant tissues and organs, and light attenuation in canopies. From an ecological perspective, all these interactions can be relevant as they could contribute to plants’ fitness, highlighting the need of multiple experimental approaches, including field experiments and multiple generations of plants, when studying phenotypic plasticity to solar radiation. In practice, only cross-disciplinary collaboration and open-minded scientific dialog will allow us to make good progress.

**OUTSTANDING QUESTIONS**

- Does UVR8 action extend into the UV-A region similarly in all plant species?
- What are the direct and indirect roles of cry-dash, PHOTs, and phytochromes inthe perception of solar UV radiation by plants?
- How does UV perception through UVR8 and CRYs affect signaling and crosstalk downstream of other plant photoreceptors?
- What are the relative contributions of the accumulation of screening pigments and protein-based signaling feedback towards the tuning of perception of different wavelength within the UV waveband?
- How does perception of solar UV radiation through photoreceptors, i.e., as an information-carrying cue, contribute to plant fitness? Do these contributions to fitness go any farther than tolerance of exposure to UV radiation itself?

## Concluding remarks

Photoreceptor-driven plant responses have been extensively studied and reported for UV-B, blue, and red/far-red spectral regions. A gap in knowledge had remained due to the lack of an equivalent research effort in the UV-A region of the solar spectrum. Recent studies showing that perception of solar UV-A_sw_ by plants is mediated by the “UV-B” photoreceptor UVR8 and the complexity of signaling interactions make it necessary to revise accepted views on the perception of solar UV radiation by plants and the role it may play in plant fitness (see Outstanding Questions). Further research is required to assess the direct and indirect roles of different photoreceptors in UV-induced changes in gene expression, morphology, growth, photosynthetic performance, and metabolite profiles. The recent studies showing that plants differentiate between UV-A_sw_ and UV-A_lw_ and differently respond to these bands serves as a base for future studies, in which it will be required to separately measure and/or manipulate UV-A_sw_ and UV-A_lw_, as we now know that in sunlight these bands are predominantly perceived through different photoreceptors.

## Supplemental data

**Supplemental Figure S1.** Commonly used UV light sources.

## Supplementary Material

kiab162_Supplementary_Data

## Notes

N.R. conceptualized the layout of the manuscript. N.R. and P.J.A. wrote the manuscript with contributions from L.O.M. All three authors revised and commented on the whole manuscript.

The author responsible for distribution of materials integral to the findings presented in this article in accordance with the policy described in the Instructions for Authors (https://academic.oup.com/plphys/pages/general-instructions) is: Neha Rai (neha.rai@unige.ch).
