# Supplementary material for: Perception of solar UV radiation by plants: photoreceptors and mechanisms
Source: Plant Physiol. 2021 Apr 7;186(3):1382–96. doi: 10.1093/plphys/kiab162 (PMC8260113; doi:10.1093/plphys/kiab162)
Supplement: kiab162_Supplementary_Data [file kiab162_supplementary_data.pdf]

NF: not filtered

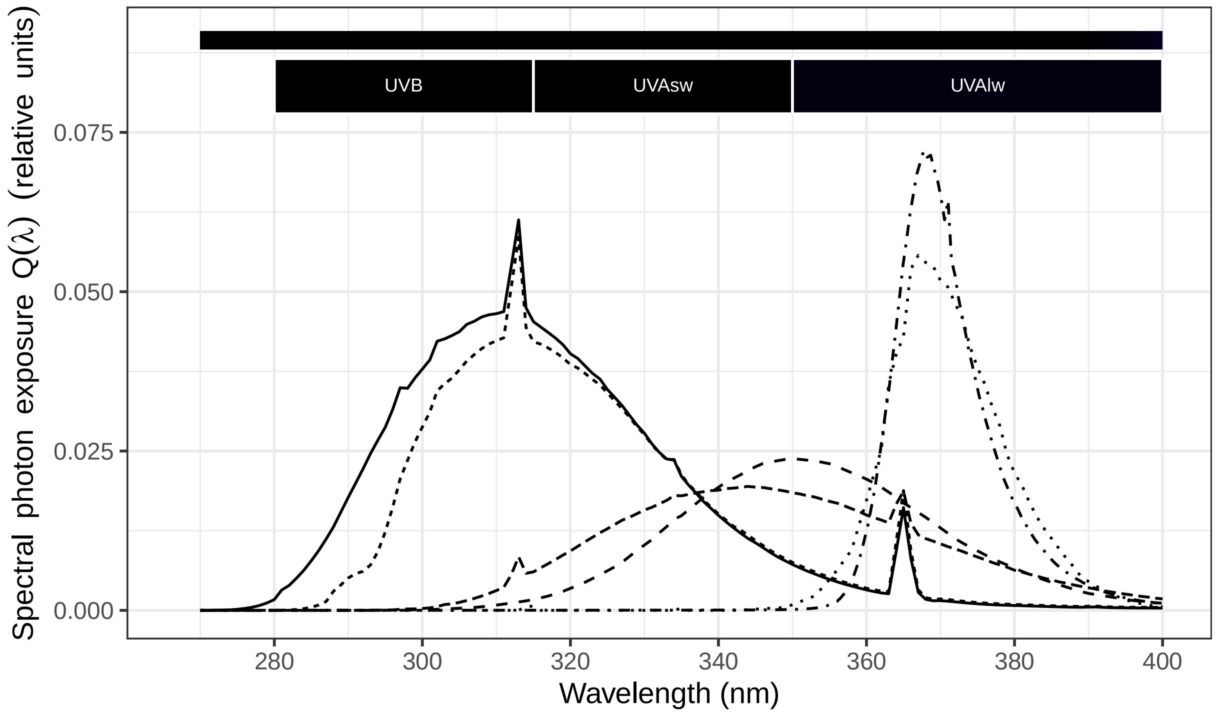

CA: filtered with cellulose acetate 0.115 mm

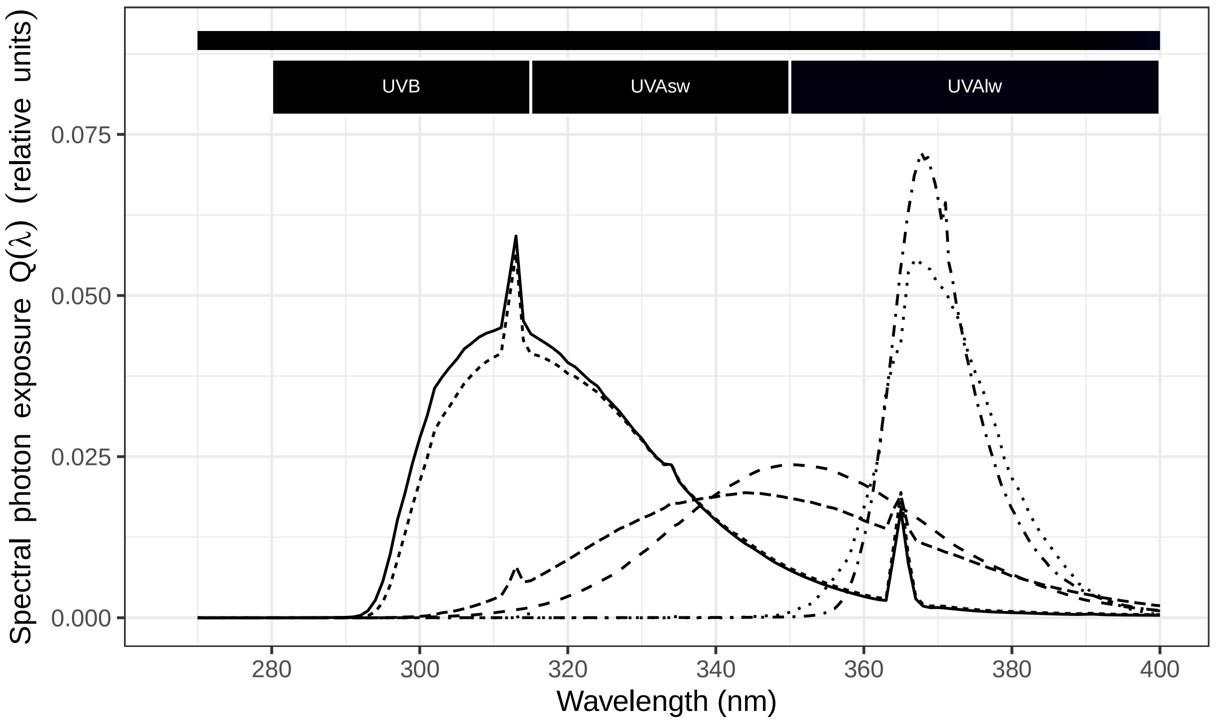

PET: filtered with polyester 0.125 mm

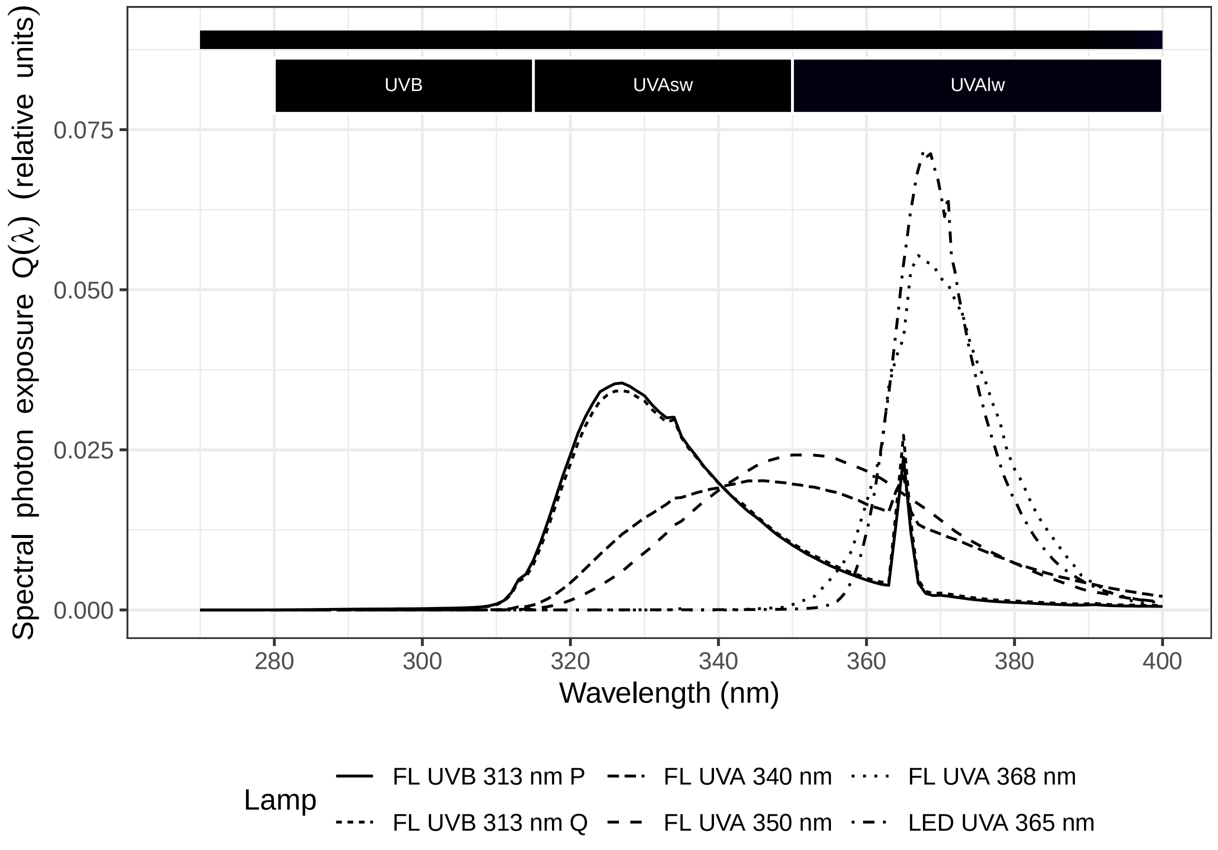

Photons per UV band (% of total UV photons)

Filter	Lamp	UV(<293nm)	UVB	UVA	UVAsw	UVAlw
NF	FL UVB 313 nm P	7	51	48	43	6
NF	FL UVB 313 nm Q	2	43	57	49	8
NF	FL UVA 340 nm	0	4	96	51	45
NF	FL UVA 350 nm	0	1	99	44	55
NF	FL UVA 368 nm	0	0	100	0	100
NF	LED UVA 365 nm	0	0	100	0	100
CA	FL UVB 313 nm P	0	42	58	51	7
CA	FL UVB 313 nm Q	0	38	62	53	9
CA	FL UVA 340 nm	0	4	96	51	46
CA	FL UVA 350 nm	0	1	99	44	55
CA	FL UVA 368 nm	0	0	100	0	100
CA	LED UVA 365 nm	0	0	100	0	100
PET	FL UVB 313 nm P	0	2	98	80	17
PET	FL UVB 313 nm Q	0	2	98	79	19
PET	FL UVA 340 nm	0	0	100	48	52
PET	FL UVA 350 nm	0	0	100	41	59
PET	FL UVA 368 nm	0	0	100	0	100
PET	LED UVA 365 nm	0	0	100	0	100

Supplemental Figure S1. Commonly used UV light sources. The measured emission spectrum of four types of light sources was convoluted with the transmittance spectra of two types of filters typically used with them. Lamps: UV-B fluorescent tubes (*FL UVB 313 nm Q:* Q-Panel UVB-313, 40W T12, Q-Lab, and *FL UVB 313 nm P:* TL12, 40W T12, Philips), UV-A fluorescent tubes (*FL UVA 340 nm:* Q-Panel UVA-340, Q-Lab; *FL UV-A 350 nm:* UV-A BLB, "similar to older F40 BLB", Light Sources, Inc.; *FL UV-A 368 nm:* TLD 36W/108 BLB, Philips), UV-A LED, nominal peak at 365 nm (LZ1-10UV00, Osram/Led Engin). Filters: CA, cellulose diacetate (0.115 mm thick, Courtaulds), PET, polyester (0.125 mm thick, Autostat CT-5, McDermit). The table shows photon ratios describing the proportion of the total UV photon irradiance contributed by waveband: UV short $\lambda < 293$ nm; UV $\lambda = 250$–400 nm; UV-B $\lambda = 280$–315 nm; UV-A_{sw} $\lambda = 315$–350 nm; and UV-A_{lw} $\lambda = 350$–400 nm. All spectra are scaled to equal UV-A ($\lambda = 315$–400 nm) photon irradiance of one. Computations were done using functions and spectral data from packages in the R for photobiology suite (Aphalo, 2015).

Literature Cited

Aphalo PJ (2015) The r4photobiology suite: spectral irradiance. UV4Plants Bull **2015 (1)**: 21–29
DOI: https://doi.org/10.19232/uv4pb.2015.1.14
